# Validation of the traditional medicinal use of a Mexican endemic orchid (*Prosthechea karwinskii*) through UPLC-ESI-qTOF-MS/MS characterization of its bioactive compounds

**DOI:** 10.1016/j.heliyon.2022.e09867

**Published:** 2022-07-06

**Authors:** Gabriela Soledad Barragán-Zarate, Luicita Lagunez-Rivera, Rodolfo Solano, Candy Carranza-Álvarez, Diego Manuel Hernández-Benavides, Gerard Vilarem

**Affiliations:** a Laboratorio de Extracción y Análisis de Productos Naturales Vegetales. Centro Interdisciplinario de Investigación para el Desarrollo Integral Regional Unidad Oaxaca, Instituto Politécnico Nacional, Hornos 1003, 71230, Oaxaca, Mexico; b Unidad Académica Multidisciplinaria de la Zona Huasteca, Universidad Autónoma de San Luis Potosí, Romualdo del Campo 501, Frac. Rafael Curiel, 79060, Ciudad Valles, San Luis Potosí, México; c Laboratoire de Chimie Agro-Industrielle, ENSIACET, 4 Allée Emile Monso, BP 44362, 31030, Toulouse, France

**Keywords:** Phenols, Flavonoids, DCFH-DA, Antioxidant, Orchid, Traditional medicine

## Abstract

**Ethnopharmacological relevance:**

The orchid *Prosthechea karwinskii* is a medicinal orchid in Oaxaca, Mexico, used to treat diabetes, cough, wounds, and burns, prevent miscarriage and assist in labor. Each part of the plant (leaves, pseudobulbs, or flowers) is used by healers for certain treatment conditions, indicating that each part has different biocompounds with specific pharmacological activity.

**Aim of the study:**

To characterize the biocompounds in extracts from leaves, pseudobulbs, and flowers of *P. karwinskii* and evaluate their ROS inhibition capacity to associate it with medicinal uses.

**Materials and methods:**

The compounds present in extracts from leaves, pseudobulbs, and flowers of *P. karwinskii* were identified by UPLC-ESI-qTOF-MS/MS. The chemical differentiation of each extract was tested by principal component analysis (PCA) using compound intensity values. For each extract, total phenol and flavonoid contents were quantified. Their antioxidant capacity was evaluated *ex vivo* by inhibition of ROS with DCFH-DA and *in vitro* with DPPH radical.

**Results:**

Based on the PCA, it was observed that some compounds were completely separated from others according to the correlation that they presented. The compounds common to all three plant parts were quinic, malic, succinic, azelaic, and pinellic acids. Among the compounds identified, two were exclusive to leaves, four to pseudobulbs, and ten to flowers. Some of the identified compounds have well-known antioxidant activity. The leaves had the highest content of total phenols and flavonoids, and the highest *in vitro* and *ex vivo* antioxidant capacity. A strong correlation was observed between phenol and flavonoid contents, and antioxidant capacity *ex vivo* and *in vitro*.

**Conclusions:**

It was found that the bioactive compounds and antioxidant capacity of each part of the plant were associated with its traditional medicinal use. A pharmacological potential was also found in *P. karwinskii* for further biological studies because of the type of compounds it contained.

## Highlights

• Compounds common and specific to each plant part were identified by UPLC-ESI-qTOF-MS/MS.
• The biological activities reported for the identified compounds validate the traditional medicinal use of *P. karwinskii*.
• Embelin (inflammation) is exclusive to leaves, phloridzin (diabetes) to pseudobulbs, and abscisic acid (in labor) to flowers.
• In the literature, the antioxidant compounds are rutin, kaempferol-3-O-rutinoside, embelin, guanosine, and azelaic acid.
• Pearson's correlation coefficients indicate that a higher content of phenols and flavonoids has a higher antioxidant capacity.

## 1 Introduction

*Prosthechea karwinskii* (Mart.) J.M.H. Shaw, an orchid endemic to southeastern Mexico (Villaseñor, 2016; Solano et al., 2020), is traditionally used in Oaxaca, Mexico. This species is appreciated and cultivated in home gardens in many villages. During Easter, people make ornaments with flowers for religious purposes, mainly to decorate Catholic churches and altars (Solano et al., 2010). In traditional medicine, different parts of the plant are used by healers for the treatment of certain conditions: leaves for the control of diabetes; pseudobulbs for diabetes, cough, and healing wounds and burns; flowers to prevent miscarriages, help in childbirth, and calm coughs (Cruz-García et al., 2014).

The health conditions for which *P. karwinskii* is used in the traditional medicine of Oaxaca are related to the inflammatory processes. Diabetes is characterized by low-grade inflammation with increased proinflammatory markers (Xu et al., 2021). Cough is an inflammation of the airway (Kamimura et al., 2010). Inflammatory cells are involved in the healing process (Wang et al., 2019). Miscarriage is associated with an inappropriate inflammatory response in pregnant women (Christiansen et al., 2006; Gao et al., 2020). In labor, there is an inflammatory overload in feto-maternal tissues (Hadley et al., 2018).

Previous studies have shown that separate extracts from the leaves, pseudobulbs, and flowers of *P. karwinskii* inhibit *in vitro* coagulation (Barragán-Zarate et al., 2016); as well as reduce glucose, triglyceride, total cholesterol, and adipose tissue in Wistar rats with metabolic syndrome (MS) induced (Rojas-Olivos et al., 2017). The extract from the leaves of this species has anti-inflammatory, gastroprotective, and inhibitory effects on reactive oxygen species (ROS) (Barragán-Zárate et al., 2020). In addition, foliar extract reduces insulin resistance, proinflammatory status, and cardiovascular risk in Wistar rats with MS-induced (Barragán-Zárate et al., 2021).

Oxidative stress can affect several physiological functions and lead to chronic diseases (Wolfe and Liu, 2007; Shen et al., 2019). ROS are a normal product of metabolism; however, when their concentration exceeds the levels that can be neutralized by endogenous antioxidant systems, oxidative stress occurs. Cell-based assays are an effective way to evaluate ROS inhibition in an *ex vivo* model. These assays quantify the bioactivity of compounds considering their adsorption, distribution, metabolism, excretion, and bioavailability (Wolfe and Liu, 2007), which ensures their bioefficacy and bioactivity (Thiengkaew et al., 2021). In contrast to chemical assays, cell-based assays reflect how the enzymes of endogenous antioxidant systems act to attenuate oxidative stress (Xu et al., 2021), so they are biologically representative of antioxidant activity (Wolfe et al., 2008; Wolfe and Liu, 2007; Shen et al., 2019). The ability of a plant to inhibit ROS (and thus oxidative stress) could be related to its therapeutic effects since oxidative stress is involved in the pathogenesis of many diseases. Therefore, a decrease in oxidative stress could be a mechanism by which the plant exerts its therapeutic effect.

Phytochemical screening of leaves, pseudobulbs, and flowers extracts from *P. karwinskii* revealed the presence of several groups of flavonoid families, phenols, anthraquinones, and saponins (Barragán-Zarate et al., 2016). Only the compounds present in leaves have been identified in extracts obtained with ultrasound assistance (Barragán-Zárate et al., 2020). However, it is unknown whether the pseudobulbs and flowers of this species have a different or similar composition to that of its leaves. However, the therapeutics used for *P. karwinskii* in traditional medicine mainly consist of the preparation of infusions (Cruz-García et al., 2014). Therefore, it is important to evaluate the extracts obtained by a preparation method similar to traditional medicine.

In traditional medicine, *P. karwinskii* is used to treat chronic degenerative diseases and other health problems related to inflammatory processes, and healers attribute specific medicinal uses to each part of the plant. This plant represents a potential source for the development of pharmaceuticals for health problems worldwide. However, the leaves are the only plant part studied for their biological activity and identification of their bioactive compounds. Our research group found that leaves have a protective effect on cardiovascular risk and regulate parameters associated with metabolic syndrome (Barragán-Zárate et al., 2021). Therefore, this study aimed to characterize the bioactive constituents present in the leaves, pseudobulbs, and flowers of *P. karwinskii* and to evaluate their ROS inhibition capacity to associate them with the medicinal use of each part of plant. We aimed to determine if the particular therapeutic properties of each plant part were due to the specific compounds they contain.

## 2 Materials and methods

### 2.1 Materials and reagents

Acetonitrile, dimethyl sulfoxide (DMSO), phosphate-buffered saline (PBS), formic acid, distilled water, ethanol, Folin-Denis reagent, quercetin, trypan blue, gallic acid, 1,1-diphenyl-2-picryl-hydrazyl (DPPH), and hydrogen peroxide (H_2_O_2_) were purchased from Sigma Aldrich (Toluca, Mexico).

### 2.2 Plant material

The vegetal material of *P. karwinskii* was rescued from discarded specimens that had been used as decorations during the Easter celebration in Zaachila, Oaxaca. To identify the plant, it was compared with a voucher specimen (Solano 4037) deposited at the OAX Herbarium of the Instituto Politécnico Nacional. The specimens were divided into pseudobulbs, leaves, and flowers, and each part was dried, ground, and sieved for compound extraction. This study was performed in accordance with Pridgeon et al. (2005), Soto et al. (2007), Villaseñor (2016), and Solano et al. (2020), in which *Prosthechea* is recognized as an accepted generic name and includes *Euchile*. Species considered within *Euchile* are included in the first category, as is the case for *E. karwisnkii* (Mart.) Christenson.

### 2.3 Soxhlet extraction

The extraction of compounds was performed using a Soxhlet apparatus following the methodology used by Barragán-Zárate et al. (2016). Therefore, 180 mL of 50% (v/v) ethanol in a water mixture was used as the solvent, and 10 g of the pulverized sample was added. The extraction was performed at 80 °C for 2 h.

### 2.4 Compound identification with UPLC-ESI-qTOF-MS/MS

To identify compounds from each plant part, we followed the method described by Barragán-Zárate et al. (2020). An ultra-high-performance liquid chromatography system (UPLC, Thermo Scientific, Ultimate 3000) combined with an Impact II mass spectrometer (Bruker) with electrospray ionization (ESI) and quadrupole time-of-flight (qTOF) was used. The column used for the analysis was a Thermo Scientific Acclaim 120 C18 (2.2 μm, 120 Å, 50 × 2.1 mm). The mobile phase used was A: 0.1% formic acid in water and B: acetonitrile, with the following gradient: 0% B (0–2 min), 1% B (2–3 min), 3% B (3–4 min), 32% B (4–5 min), 36% B (5–6 min), 40% B (6–8 min), 45% B (8–9 min), 80% B (9–11 min), 0% B (12–14). The flow rate was 0.35 mL/min, and the injection temperature was 25 °C. The analysis was performed using autoMSMS. The mass spectrometer was operated in negative electrospray mode at 0.4 bar (5.8 psi) over the mass range of 50–700 m/z. The capillary voltage ionization (Vcap) was 2700 V, and diode array detector (DAD) analysis was performed in the range of 200–800 nm and stored at 280 and 255 nm. The data obtained were processed using DataAnalysis software.

The main compounds in each plant part were identified by comparing their exact mass and MS/MS spectra with those reported in the MetaboBase libraries of Bruker and Massbank and those previously reported in scientific articles.

### 2.5 Determination of total phenols and flavonoids content

The total phenol content was determined using the Folin-Denis reagent according to the procedure described by Swain and Hillis (1959). Therefore, the extract of each plant part was prepared at a concentration of 5 mg mL^−1^. Gallic acid was used as a reference standard, and the results were expressed as mg gallic acid equivalents per gram of sample (mg GAE g-1).

Total flavonoid content was determined as reported by Chang et al. (2002). Each extract was prepared at a concentration of 5 mg mL^−1^. Quercetin was used as a reference standard, and the results were expressed as mg quercetin equivalents per gram of sample (mg QueE g-1).

### 2.6 Inhibition of ROS in an *ex vivo* model cell-based

To measure the antioxidant capacity of the extracts at the cellular level, the DCFH-DA reagent was used to measure the ROS in the cells. Upon entering the cells and reacting with the ROS, it oxidizes and produces 2,7-dichlorofluorescein (DCF), a fluorescent compound whose intensity is directly proportional to the amount of ROS present in cells (Torres-Rodríguez et al., 2016). Therefore, peripheral blood mononuclear cells (PBMC) were used following the method described by Barragán-Zárate et al. (2020). In a 96-well plate, 1 × 10^5^ cells were suspended in 100 μL of RPMI medium, and 1.22 μL of DCFH-DA (4.10 mM) was added to each well. Subsequently, 50 μL of each extract was added at a concentration of 1000 μg of extract/mL in 0.1% DMSO. The well to which no extract was added was considered the positive control. The plates were incubated for 30 min at 37 °C, with 5% carbon dioxide (CO_2_) and 95% humidity. Subsequently, 100 μL of PBS was added to each well, centrifuged at 1500 rpm for 5 min at 25 °C, and decanted to remove the supernatant. Thereafter, 100 μL of H_2_O_2_ (350 μM) dissolved in RPMI medium was added to each well and incubated for 30 min at 37 °C, with 5% CO_2_ and 95% humidity for 5 h. The results are expressed as the percentage inhibition of ROS relative to the positive control.

### 2.7 Determination of *in vitro* antioxidant capacity

The *in vitro* antioxidant capacity of each extract was determined using the 1,1-diphenyl-2-picrylhydrazyl (DPPH) free radical scavenging method, as described by Scherer and Godoy (2009). Extracts were prepared at a concentration of 4 mg/mL. The results are expressed as % inhibition relative to the negative control (DPPH with methanol and no extract).

### 2.8 Data processing and statistical analysis

Data for total phenol and flavonoid contents, as well as *in vitro* and cell antioxidant capacity, are presented as mean ± standard deviation (SD). An analysis of variance (ANOVA), which compares different means, was performed, followed by a Tukey’s means comparison test, both implemented in Graph Pad 5.0. Differences were considered statistically significant at p < 0.05. In addition, a correlation test between total phenols, flavonoid content, and antioxidant capacity *in vitro* (DPPH radical inhibition) and *ex vivo* (ROS inhibition) was performed using the Pearson correlation coefficient.

The raw data files from UPLC-ESI-qTOF-MS/MS analysis of leaves, pseudobulbs, and flowers of *P. karwinskii* were collected using DataAnalysis (Bruker) and imported into MetaboScape 3.0 (Bruker) for data processing, including peak extraction. The retention time (TR), m/z, and peak intensity were obtained for each compound. Principal component analysis (PCA) and cluster analysis with unweighted pair group method with arithmetic mean (UPGMA) were applied to the var-covar matrix of intensity values, for which the ordination of the compounds by plant part based on their similarities was evaluated. PCA and cluster analysis were performed using the PAST 4.09 software (Hamer, 2022).

## 3 Results

### 3.1 Compounds identified in leaves, pseudobulbs, and flowers extracts of *Prosthechea karwinskii*

**Figure 1:**
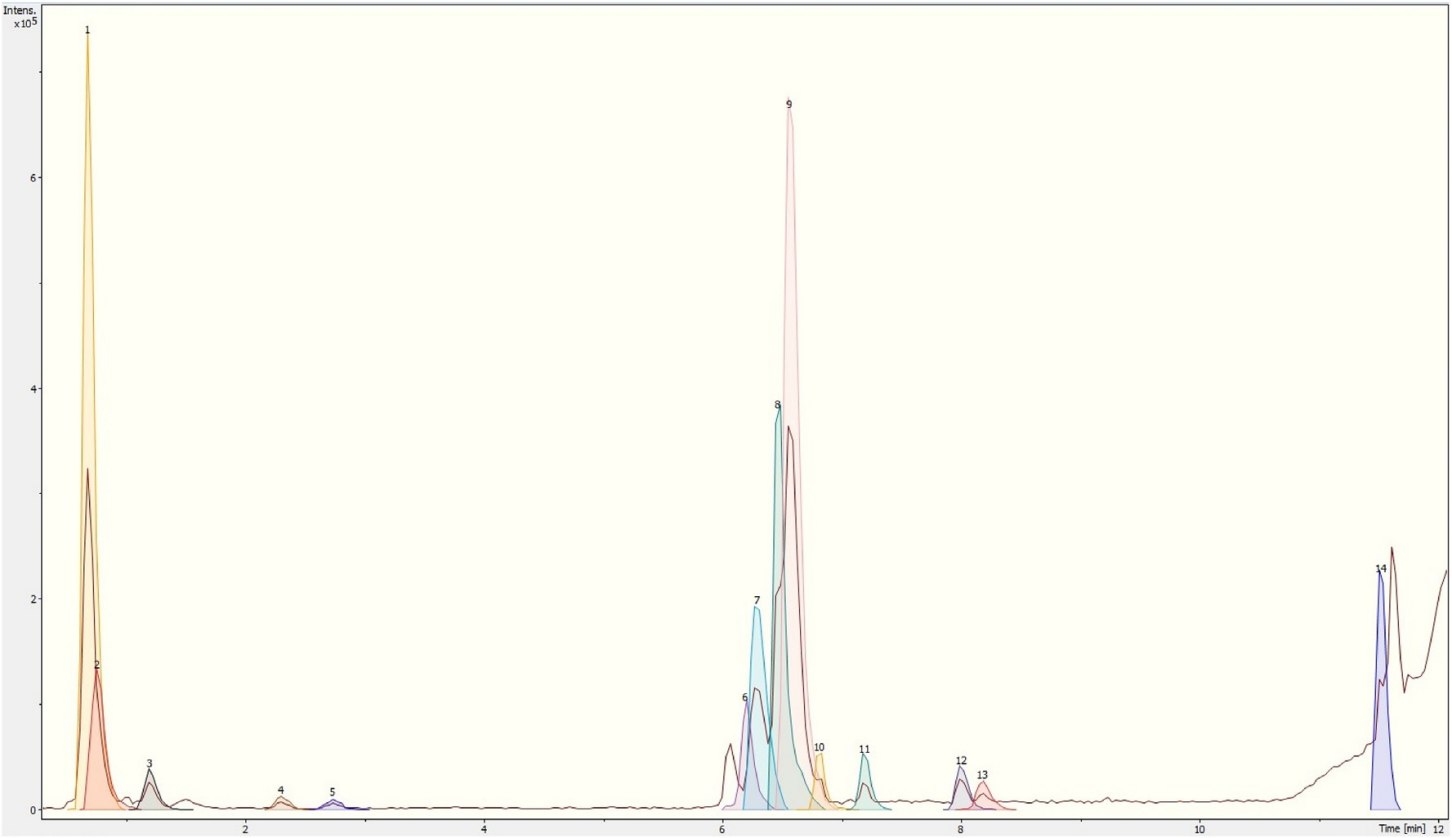
Chromatogram of *Prosthechea karwinskii* leaves extract obtained with UPLC-ESI-qTOF-MS/MS. 1 (Quinic acid), 2 (Malic acid), 3 (Succinic acid), 4 (L-(-)-phenylalanine), 5 (Guanosine), 6 (Neochlorogenic acid), 7 (Chlorogenic acid), 8 (Rutin), 9 (Kaempferol-3-O-rutinoside), 10 (Azelaic acid), 11 (Sebacic acid), 12 (N-undecanoylglycine), 13 (Pinellic acid), 14 (embelin).

**Figure 2:**
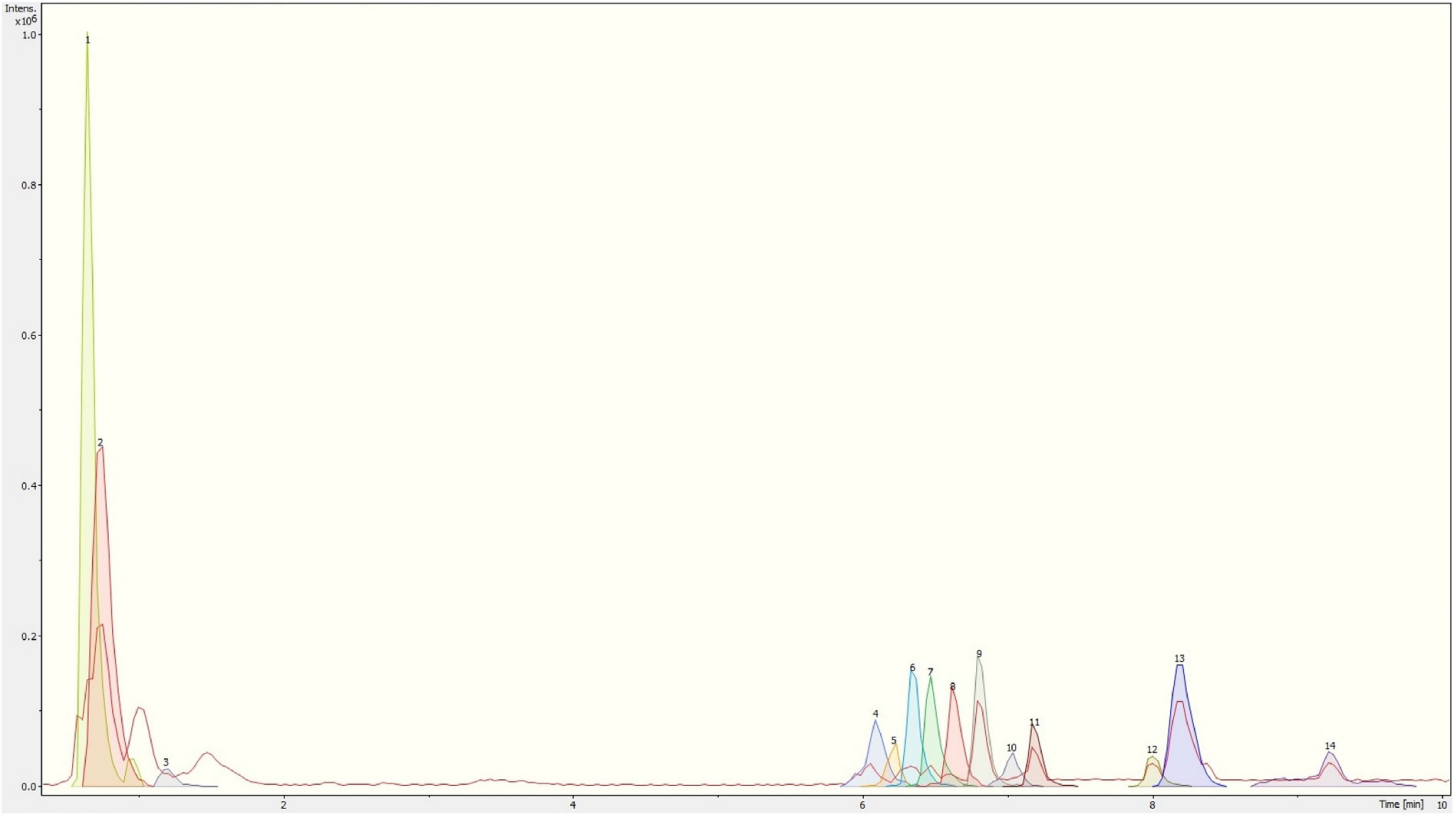
Chromatogram of *Prosthechea karwinskii* pseudobulbs extract obtained with UPLC-ESI-qTOF-MS/MS. 1 (Quinic acid), 2 (Malic acid), 3 (Succinic acid), 4 (3-Methylglutaric acid), 5 (Neochlorogenic acid), 6 (Chlorogenic acid), 7 (Rutin), 8 (2-Hydroxysebacic acid), 9 (Azelaic acid), 10 (Phloridzin), 11 (Sebacic acid), 12 (N-undecanoylglycine), 13 (Pinellic acid), 14 (Gibberellin A7).

**Figure 3:**
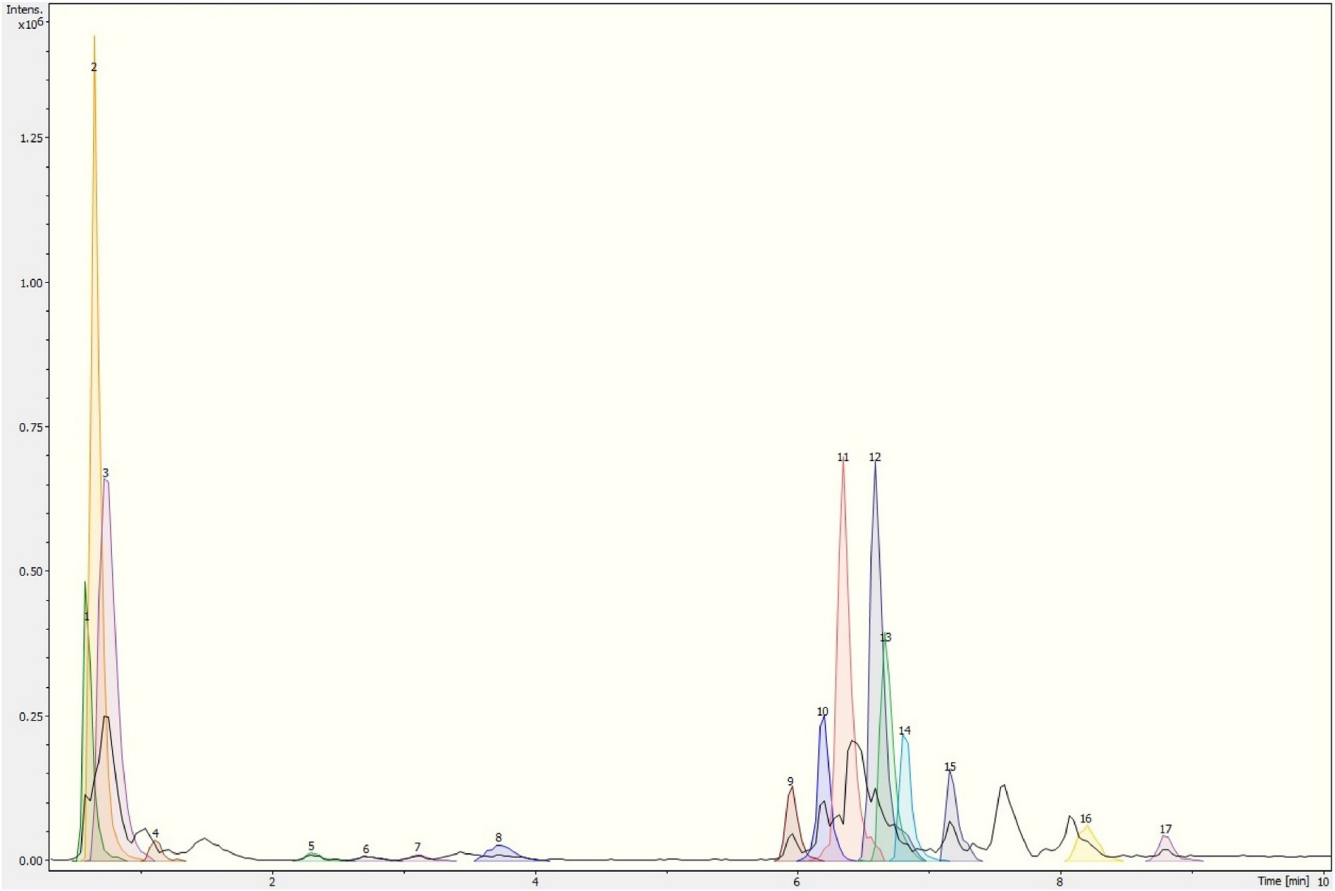
Chromatogram of *Prosthechea karwinskii* flowers extract obtained with UPLC-ESI-qTOF-MS/MS. 1 (D-Tagatose), 2 (Quinic acid), 3 (Malic acid), 4 (Succinic acid), 5 (L-(-)-Phenylalanine), 6 (Guanosine), 7 (1,3,4, 6-tetra-O-acetyl-2-deoxyhexopyranose), 8 (1-O-salicyl-D-glucose), 9 (1-O-vanillyl-beta-D-glucose), 10 (Calaliukiuenoside), 11 (2-Methyl-2-propanoyl 2,3,4,6-tetra-O-acetyl-D-glucopyranoside), 12 ((+)-abscisic acid β-D-glucopyranosyl ester), 13 (Myricitrin-5-methyl ether), 14 (Azelaic acid), 15 (Abscisic acid), 16 (Pinellic acid), 17 ((1R,3S,4R)-1-[(3R-4S-6R)-3,4,5,6-tetrahydroxy-6-methoxy-hexoxy)] hexane-1,2,3,4,6-pentol).

Figures 1, 2, and 3 show the UPLC-ESI-qTOF-MS/MS chromatograms from the leaves, pseudobulbs, and flowers extracts of *P. karwinskii*, respectively. The corresponding lists of compounds identified are given in Tables 1, 2, and 3. Among the identified compounds, quinic, malic, succinic, azelaic, and pinellic acids were found in all three parts of the plant. Neochlorogenic, chlorogenic, and sebacic acids, as well as rutin and N-undecanoylglycine, were found in both the leaves and pseudobulbs. Leaves and flowers share compounds, such as L-(-)-phenylalanine and guanosine.

**Table 1:** Identification of compounds in the leaves extract from *Prosthechea karwinskii* with UPLC-ESI-qTOF-MS/MS.

Peak	RT(min)	m/z [M-H]^-^	Error (ppm)	MS/MS fragments	Compound (Chemical formula)	Chemical structure
1	0.7	191.0557	1.9	85.0293, 87.0078, 111.0443, 127.6945	Quinic Acid^c^ (C_7_H_12_O_6_)	
2	0.8	133.0140	1.8	115.0032	Malic acid^c^ (C_4_H_6_O_5_)	
3	1.2	117.0191	2.3	73.0290, 99.0072	Succinic acid^c^ (C_4_H_6_0_4_)	
4	2.3	164.0712	3.2	72.0072, 103.0539, 147.0442	L-(-)-Phenylalanine^d^ (C_9_H_11_NO_2_)	
5	2.7	282.0833	3.9	108.5347, 133.0157, 150.0429	Guanosine^d^ (C_10_H_13_N_5_O_5_)	
6	6.0	353.0867	3.1	173.0430, 179.0365, 191.0556	Neochlorogenic acid^b^ (C_16_H_18_O_9_)	
7	6.3	353.0866	3.4	173.0452, 179.0365, 191.0556	Chlorogenic acid^a^^,^^b^ (C_16_H_18_O_9_)	
8	6.5	609.1438	3.8	300.0266, 301.0335	Rutin^a^^,^^d^ (C_27_H_30_O_16_)	
9	6.6	593.1489	3.9	284.0314, 285.0393	Kaempferol-3-O-rutinoside^d^ (C_27_H_30_O_15_)	
10	6.8	187.0970	3.1	97.0653, 125.0963, 169.0889	Azelaic acid^e^ (C_9_H_16_0_4_)	
11	7.2	201.1127	2.4	139.1128, 183.1021	Sebacic acid^e^ (C_10_H_18_O_4_)	
12	8.0	242.1756	2.2	181.1601, 225.1495	N-undecanoylglycine^e^ (C_13_H_25_NO_3_)	
13	8.2	329.2321	3.9	171.1023, 229.1436	Pinellic acid^e^ (C_18_H_34_O_5_)	
14	11.5	293.2112	3.6	223.1685, 235.1680, 275.2013	Embelin^e^ (C_17_H_26_O_4_)	

Superscripts indicate the support reference for identifying the compound. RT: Retention time.

a De Souza et al. (2016).

b Liu et al. (2016).

c MassBank library.

d Bruker's MetaboBase library.

e CompoundCrawler.

**Table 2:** Identification of compounds in the pseudobulbs extract from *Prosthechea karwinskii* with UPLC-ESI-qTOF-MS/MS.

Peak	RT (min)	m/z [M-H]^-^	Error (ppm)	MS/MS fragments	Compound (Chemical formula)	Chemical structure
1	0.7	191.0557	4.6	85.0293, 127.0396	Quinic Acid^c^ (C_7_H_12_O_6_)	
2	0.8	133.0140	3.6	115.0032	Malic acid^d^ (C_4_H_6_O_5_)	
3	1.2	117.0191	1.5	73.0290, 99.0072	Succinic acid^c^ (C_4_H_6_0_4_)	
4	6.1	145.0502	7.4	83.0491, 101.0606	3-Methylglutaric acid^d^ (C_6_H_10_O_4_)	
5	6.0	353.0867	4.3	173.0430, 179.0365, 191.0556	Neochlorogenic acid^b^ (C_16_H_18_O_9_)	
6	6.3	353.0866	4.3	173.0452, 179.0365, 191.0556	Chlorogenic acid^a^^,^^b^^,^^d^ (C_16_H_18_O_9_)	
7	6.5	609.1438	4.9	300.0266, 301.0335	Rutin^d^ (C_27_H_30_O_16_)	
8	6.6	217.1074	2.8	155.1086, 171.1025, 199.0976	2-Hydroxysebacic acid^d^ (C_10_H_18_O_5_)	
9	6.8	187.0970	3.4	125.0967	Azelaic acid^e^ (C_9_H_16_0_4_)	
10	7.0	435.1639	4.2	273.1122	Phloridzin^d^ (C_21_H_24_O_10_)	
11	7.2	201.1127	4.1	139.1124, 183.1017	Sebacic acid^e^ (C_10_H_18_O_4_)	
12	8.0	242.1756	3.9	181.1588, 225.1468	N-undecanoylglycine^e^ (C_13_H_25_NO_3_)	
13	8.2	329.2321	4.7	171.1020, 211.1336	Pinellic acid^e^ (C_18_H_34_O_5_)	
14	9.2	329.1379	4.2	223.0939	Gibberellin A7^c^ (C_19_H_22_O_5_)	

Superscripts indicate the support reference for identifying the compound. RT: Retention time.

a De Souza et al. (2016).

b Liu et al. (2016).

c MassBank library.

d Bruker’s MetaboBase library.

e CompoundCrawler.

**Table 3:** Identification of compounds in the flowers extract from *Prosthechea karwinskii* with UPLC-ESI-qTOF-MS/MS.

Peak	RT (min)	m/z [M-H]^-^	Error (ppm)	MS/MS fragments	Compound (Chemical formula)	Chemical structure
1	0.65	179.0555	3.7	89.0245, 101.0238	D-Tagatose^b^ (C_6_H_12_O_6_)	
2	0.7	191.0557	3.3	85.0293, 87.0073, 111.0084, 127.0387	Quinic Acid^a^ (C_7_H_12_O_6_)	
3	0.8	133.0140	3.1	115.0032	Malic acid^a^ (C_4_H_6_O_5_)	
4	1.2	117.0191	4.1	73.0290, 99.0072	Succinic acid^a^ (C_4_H_6_O_4_)	
5	2.3	164.0712	4.4	72.0072, 103.0539, 147.0442	L-(-)-Phenylalanine^b^ (C_9_H_11_NO_2_)	
6	2.7	282.0833	3.8	108.5347, 133.0157, 150.0429	Guanosine^b^ (C_10_H_13_N_5_O_5_)	
7	3.1	331.1025	2.9	105.0343, 123.0430, 267.0969	1,3,4,6-tetra-O-acetyl-2-deoxyhexopyranose^c^ (C_14_H_20_O_9_)	
8	3.7	299.0762	3.4	93.0338, 137.0243	1-O-salicyl-D-glucose^c^ (C_13_H_16_O_8_)	
9	6.0	329.0866	3.7	123.0446, 152.0094 167.0346	1-O-vanilloyl-beta-D-glucose^c^ (C_14_H_18_O_9_)	
10	6.2	451.2170	3.3	119.0345, 167.1080, 179.0547, 405.2123	Calaliukiuenoside^c^ (C_20_H_36_O_11_)	
11	6.4	403.1595	3.7	197.1175, 241.1073	2-Methyl-2-propanyl 2,3,4,6-tetra-O-acetyl-D-glucopiranoside^c^ (C_18_H_28_O_10_)	
12	6.6	425.1800	4.0	153.0906, 263.1288	(+)-abscisic acid β-D-glucopyranosyl ester^c^ (C_21_H_30_O_9_)	
13	6.7	477.1019	4.1	314.0424	Myricitrin-5-methyl ether^c^ (C_22_H_22_O_12_)	
14	6.8	187.0970	3.7	97.0644, 125.0967, 169.0880	Azelaic acid^c^ (C_9_H_16_O_4_)	
15	7.2	263.1278	4.0	163.0755, 204.1138, 219.1388	Abscisic acid^b^ (C_15_H_20_O_4_)	
16	8.2	329.2321	4.4	171.1020, 211.1327, 229.1424	Pinellic acid^c^ (C_18_H_34_O_5_)	
17	8.8	359.2063	4.5	317.1943	(1R,3S,4R)-1-[(3R-4S-6R)-3,4,5,6-tetrahydroxy-6-methoxy-hexoxy] hexane-1,2,3,4,6-pentol^a^ (C_13_H_28_O_11_)	

Superscripts indicate the support reference for identifying the compound. RT: Retention time.

a MassBank library.

b Bruker's MetaboBase library.

c CompoundCrawler.

There are also compounds unique to each part of *P. karwinskii*. Embelin and kaempferol-3-O-rutinoside were only identified in the extract obtained from the leaves (Figure 1, Table 1). Compounds such as 3-methylglucaric acid, 2-hydroxysebacic acid, phloridzin, and gibberellin A7 were identified only in the extract from the pseudobulbs (Figure 2, Table 2). The compounds identified only in the flowers extract were D-tagatose, 1,3,4,6-tetra-O-acetyl-2-deoxyhexopyranose, 1-O-salicyl-D-glucose, 1-O-vanilloyl-beta-D-glucose, calaliukiuenoside, 2-Methyl-2-propanoyl 2,3,4,6-tetra-O-acetyl-D-glucopyranoside, (+)-abscisic acid β-D-glucopyranosyl ester, myricitrin-5-methyl ether, abscisic acid, and (1R,3S,4R)-1-[(3R-4S-6R)-3,4,5,6-tetrahydroxy-6-methoxy-hexoxy] hexane-1,2,3,4,6-pentol (Figure 3, Table 3).

**Figure 4:**
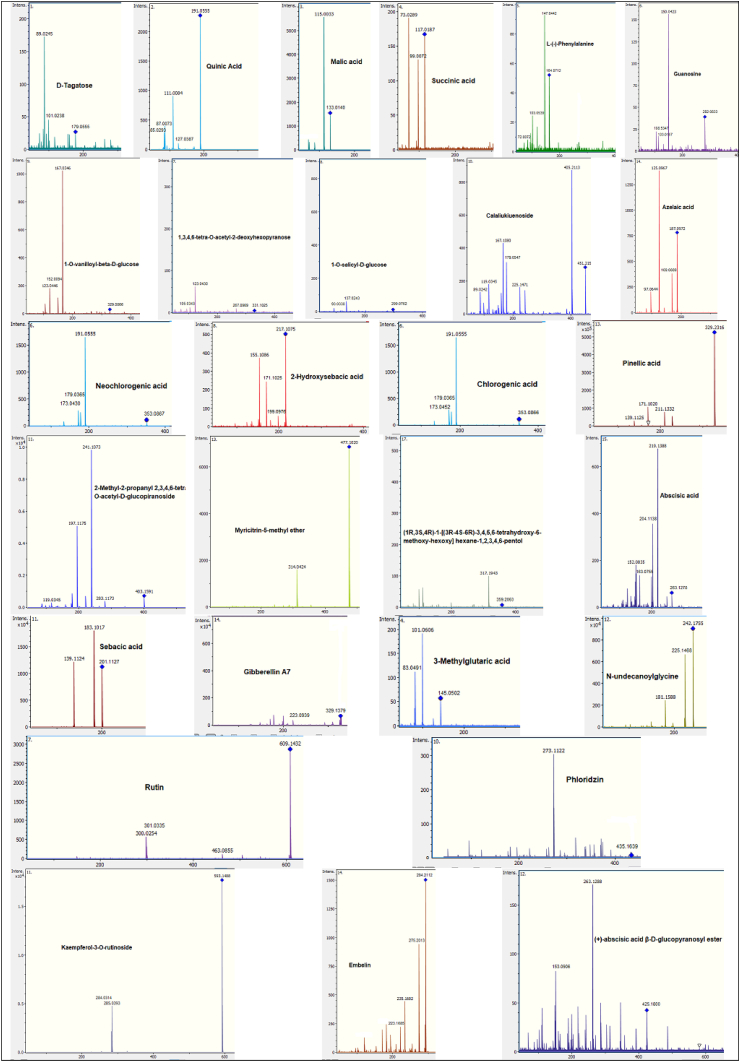
MS/MS spectra of the compounds identified in leaves, pseudobulbs, and flowers extracts of *Prosthechea karwinskii*.

Figure 4 shows the MS/MS spectra of all the compounds identified in the leaves, pseudobulbs, and flowers of *P. karwinskii*. These fragmentation patterns were used to identify the compounds by comparison with previously published libraries and the literature.

### 3.2 Principal component analysis based on UPLC-ESI-qTOF-MS/MS of leaves, pseudobulbs, and flowers of *Prosthechea karwinskii*

**Figure 5:**
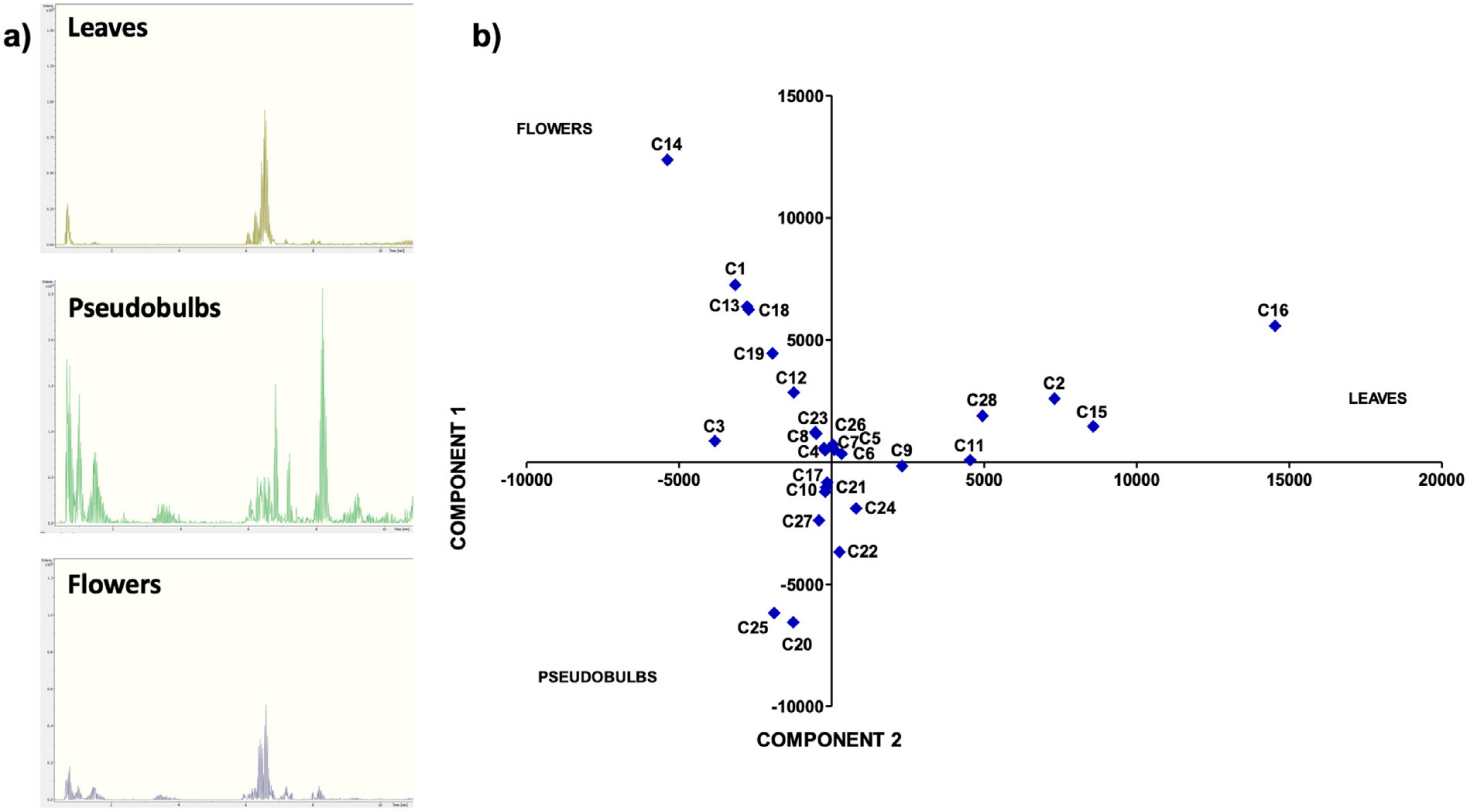
Total ion current chromatograms of leaves, pseudobulbs, and flowers of *Prosthechea karwinskii* (a). Principal Component Analysis (b). C1 = D-Tagatose, C2 = Quinic Acid, C3 = Malic acid, C4 = Succinic acid, C5 = L-(-)-Phenylalanine, C6 = Guanosine, C7 = 1,3,4,6-tetra-O-acetyl-2-deoxyhexopyranose, C8 = 1-O-salicyl-D-glucose, C9 = Neochlorogenic acid, C10 = 3-Methylglutaric acid, C11 = Chlorogenic acid, C12 = 1-O-vanilloyl-beta-D-glucose, C13 = Calaliukiuenoside, C14 = 2-Methyl-2-propanyl 2,3,4,6-tetra-O-acetyl-D-glucopiranoside, C15 = Rutin, C16 = Kaempferol-3-O-rutinoside, C17 = 2-Hydroxysebacic acid, C18 = (+)-abscisic acid β-D-glucopyranosyl ester, C19 = Myricitrin-5-methyl ether, C20 = Azelaic acid, C21 = Phloridzin, C22 = Sebacic acid, C23 = Abscisic acid, C24 = N-undecanoylglycine, C25 = Pinellic acid, C26 = (1R,3S,4R)-1-[(3R-4S-6R)-3,4,5,6-tetrahydroxy-6-methoxy-hexoxy] hexane-1,2,3,4,6-pentol, C27 = Gibberellin A7, C28 = Embelin.

The total ionic current (TIC) chromatograms from UPLC-ESI-qTOF-MS/MS analyses of the leaves, pseudobulbs, and flowers of *P. karwinskii* are shown in Figure 5a, and the PCA graph is shown in Figure 5b. The differences between the TIC chromatograms of the different parts of the plant can be observed with the naked eye (Figure 5a). For PCA components 1 and 2 accumulated 100% of the variance (79.867% and 20.133%, respectively). The compounds with the highest contribution to Component 1 were kaempferol-3-O-rutinoside (0.67092), rutin (0.39572), quinic acid (0.33716), embelin (0.22808), and chlorogenic acid (0.20955). In contrast, the compounds with the highest contribution to Component 2 were 2-Methyl-2-propanyl 2,3,4,6-tetra-O-acetyl-D-glucopiranoside (0.57137), D-tagatose (0.33499), and azelaic acid (−0.30245). Several compounds aggregated in the central part of the PCA graph because their correlation values were very low. It can be observed that there are also points completely separated from the central part, they correspond to compounds with higher correlation values.

**Figure 6:**
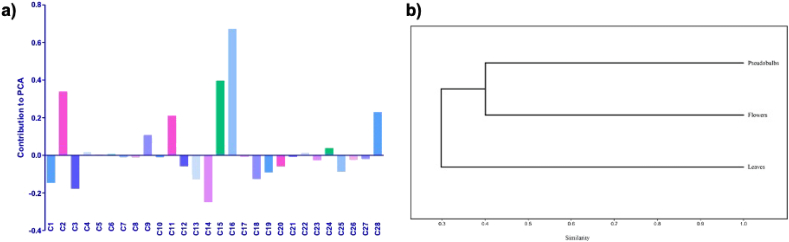
Graph of the contribution of *Prosthechea karwinskii* leaves, pseudobulbs, and flowers compounds to the components of PCA (a). Dendrogram obtained using the UPGMA method for the var-covar matrix of intensity values of compounds (b). C1 = D-Tagatose, C2 = Quinic Acid, C3 = Malic acid, C4 = Succinic acid, C5 = L-(-)-Phenylalanine, C6 = Guanosine, C7 = 1,3,4,6-tetra-O-acetyl-2-deoxyhexopyranose, C8 = 1-O-salicyl-D-glucose, C9 = Neochlorogenic acid, C10 = 3-Methylglutaric acid, C11 = Chlorogenic acid, C12 = 1-O-vanilloyl-beta-D-glucose, C13 = Calaliukiuenoside, C14 = 2-Methyl-2-propanyl 2,3,4,6-tetra-O-acetyl-D-glucopiranoside, C15 = Rutin, C16 = Kaempferol-3-O-rutinoside, C17 = 2-Hydroxysebacic acid, C18= (+)-abscisic acid β-D-glucopyranosyl ester, C19 = Myricitrin-5-methyl ether, C20 = Azelaic acid, C21 = Phloridzin, C22 = Sebacic acid, C23 = Abscisic acid, C24 = N-undecanoylglycine, C25 = Pinellic acid, C26 = (1R,3S,4R)-1-[(3R-4S-6R)-3,4,5,6-tetrahydroxy-6-methoxy-hexoxy] hexane-1,2,3,4,6-pentol, C27 = Gibberellin A7, C28 = Embelin.

Figure 6a shows the contribution of all compounds from the leaves, pseudobulbs, and flowers of *P. karwinskii* to components 1 and 2 of the PCA; positive and negative scores indicate the type of association with the variance of the components. Figure 6b shows a dendrogram of the similarities among parts of the plant based on their compounds, which was performed to assess whether clusters or differentiations were generated from the compounds. The dendrogram joins pseudobulbs and flowers extracts because of their similar compounds and separates them from the leaves extract.

### 3.3 Total phenols, flavonoids content, antioxidant capacity, and their correlation coefficients in leaves, pseudobulbs, and flowers extracts of *Prosthechea karwinskii*

Table 4 shows the phenol and flavonoid contents, antioxidant capacity, and correlation coefficients in leaves, pseudobulbs, and flowers extracts of *P. karwinskii*. The leaves extract had the highest total phenol and flavonoid contents, followed by pseudobulbs and flowers extracts.

**Table 4:** Total phenols and flavonoids content, antioxidant capacity, and their correlation coefficients in leaves, pseudobulbs, and flowers extracts of *Prosthechea karwinskii.*

Part of plant	Total phenols (mg GAE g^−1^)	Total flavonoids (mg QueE g^−1^)	ROS inhibition (%)	DPPH radical inhibition (%)
Leaves	142.95 ± 5.73^a^	11.24 ± 0.59^a^	74.56 ± 11.36^a^	56.61 ± 2.21^a^
Pseudobulbs	25.32 ± 2.83^b^	0.57 ± 0.16^b^	65.83 ± 7.52^a^	17.29 ± 1.17^c^
Flowers	22.06 ± 4.77^b^	0.22 ± 0.05^b^	53.08 ± 9.70^b^	12.86 ± 1.15^b^
Pearson correlation coefficient				
Total phenols		0.999	0.821	0.997
Total flavonoids			0.823	0.998
ROS inhibition				0.858

mg GAE g^−1^: mg gallic acid equivalent per g of sample; mg QueE g^−1^: mg quercetin equivalent per g of sample; ROS: reactive oxygen species, DPPH: 1,1-diphenyl-2-picryl-hydrazyl. Results are shown as the mean ± standard deviation. Superscripts with different letters indicate significant differences (P ≤ 0.05).

From the total content of phenolics and flavonoids quantified, the phenolic compounds identified in *P. karwinskii* (Tables 1, 2, and 3) were neochlorogenic acid and chlorogenic acid, which were present both in leaves and pseudobulbs, whereas the flavonoids identified were Kaempferol-3-rutinoside (only in leaves), rutin (in leaves and pseudobulbs), phloridzin (only in pseudobulbs), and miricitrin-5-methyl ether (only in flowers).

The antioxidant capacity (*ex vivo* by inhibition of ROS in cells and *in vitro* by inhibition of DPPH radicals) of leaves, pseudobulbs, and flowers extracts. Leaves and pseudobulbs extracts showed the highest ROS inhibition in cells, whereas the flowers extract exhibited the lowest. Regarding the inhibition of DPPH radicals, the leaves extract showed the highest activity, followed by the pseudobulbs and flowers extracts. A strong correlation was observed between the flavonoids and total phenol content (0.999) of each plant part. There were strong positive correlations between flavonoids and total phenols with the ability to inhibit DPPH radicals (0.998 and 0.997, respectively), as well as between flavonoids and total phenols with the ability to inhibit ROS (0.823 and 0.821, respectively) (Table 4).

## 4 Discussion

### 4.1 Compounds present in leaves, pseudobulbs, and flowers of *Prosthechea karwinskii* and its relationship with the orchid’s ethnopharmacological properties

The compounds identified here may be responsible for the medicinal properties of *P. karwinskii* (Cruz-García et al., 2014). Leaves and pseudobulbs are used to treat diabetes (Cruz-García et al., 2014). The possible compounds responsible for this property, identified in both plant parts, are chlorogenic acid and rutin (Ong et al., 2013; Ma et al., 2015; Ghorbani et al., 2017). Chlorogenic acid improves glucose uptake by skeletal muscles, decreases serum glucose levels (Ong et al., 2013), and decreases insulin resistance (Ong et al., 2013; Ma et al., 2015). Rutin improves the glycemic status in diabetes by decreasing small intestinal carbohydrate absorption, inhibiting tissue gluconeogenesis, increasing tissue glucose uptake, stimulating insulin secretion from β-cells, and protecting islets of Langerhans degeneration (Ghorbani et al., 2017). Other compounds with antidiabetic activity were embelin and phloridzin. Embelin, present only in leaves extract, increases antioxidant enzyme activity and regenerates pancreatic β-cell islets of Langerhans (Gupta et al., 2012). Phloridzin, which is present only in pseudobulbs extract, reduces blood glucose levels in mice (Kobori et al., 2012). These compounds could confer *P. karwinskii* its pharmacological properties related to diabetes treatment by acting alone or synergistically.

The medicinal uses of *P. karwinskii* are also related to the inflammatory processes. Quinic acid with inflammation-related activity was identified in all three extracts. This compound inhibits the nuclear factor-kappa light chain of activated β-cell (NF-κβ) and mitogen-activated protein kinase (MAP kinase) signaling pathways (Jang et al., 2017). Compounds found in both leaves and pseudobulbs, such as chlorogenic acid, rutin, and neochlorogenic acid, in addition to inhibiting MAP kinase and NF-κβ signaling, can inhibit nitric oxide (NO) production, decrease the expression of cyclooxygenase-2 (COX-2) and inducible nitric oxide synthase (iNOS), and decrease the production of proinflammatory cytokines (Hwang et al., 2014; Wang et al.*,* 2012; Yeh et al., 2014; Kim et al., 2015; Young et al., 2018; Zhao et al., 2020). Embelin, present only in leaves extract, can decrease the production of the proinflammatory cytokines interleukin 1 beta (IL-1β), tumor necrosis factor-α (TNF-α), and interleukin 6 (IL-6) (Kumar et al., 2011; Naik et al., 2013). Guanosine, found in leaves and flowers, decreases the expression of iNOS and proinflammatory cytokines TNF-α and IL-1β (Quincozes-Santos et al., 2014). These compounds, acting alone or synergistically, may be responsible for the ability of the plant to treat inflammation-related disorders.

The remedy to prevent miscarriages and help during labor is an infusion with flowers adding a coin or gold jewel, or in some cases, a potter wasp (Cruz-García et al., 2014). Gold-derived compounds have shown efficiency in treating inflammatory diseases such as rheumatoid arthritis (Ott, 2009; Cuevas et al., 2012); gold nanoparticles can enhance the anti-inflammatory properties of compounds such as chlorogenic acid through gold reduction by OH^−^ oxidation from the caffeic acid moiety present (Hwang et al., 2015). In addition, it can decrease the expression of proinflammatory markers (Muller et al., 2017). Abscisic acid, which is only found in flowers extract, can increase the expression of genes involved in muscle relaxation of the blood vessel wall (Guri et al., 2010). Because of this property, this compound could be related to the use of flowers to prevent miscarriage and aid in labor. Abscisic acid also has an antinociceptive effect (Mollashahi et al., 2018), which could facilitate labor. Potter wasps sometimes added to the flower’s infusion (Cruz-García et al., 2014) may decrease pain, as some compounds present in wasp venom have been reported to have antinociceptive effects (Gonçalves et al., 2016; Monge-Fuentes et al., 2018).

### 4.2 Phenols and flavonoids in extracts of *Prosthechea karwinskii* leaves, pseudobulbs, and flowers

Phenolic compounds and flavonoids are known for their biological properties similar to those of *P. karwinskii* in traditional medicine, as they may exert a protective effect against diabetes (Varshney et al., 2019; Salau et al., 2020; Dinda et al., 2020; Hussain et al., 2020), have anti-inflammatory activity (Bayat et al., 2021; Liskova et al., 2021) and aid in wound healing (Carvalho et al., 2021). Therefore, these compounds might be responsible for the pharmacological properties of *P. karwinskii*.

Antioxidant is another activity presented by phenols and flavonoids. They trap ROS and inhibit the action of enzymes or trace elements involved in free radical generation (Banafsheh and Sirous, 2016). According to Pearson's correlation coefficients, the presence of a higher content of phenols and flavonoids in the leaves of *P. karwinskii* is related to a higher antioxidant capacity in this part of the plant than in the pseudobulbs and flowers.

### 4.3 Antioxidant capacity of *Prosthechea karwinskii* leaves, pseudobulbs, and flowers

*Prosthechea karwinskii* extracts have antioxidant capacity both *in vitro* and *ex vivo*; therefore, they can treat ailments affecting human health caused by oxidative stress. Antioxidant compounds protect organisms from damage caused by free radicals that induce oxidative stress (Ozsoy et al., 2008). Health problems related to oxidative stress include diabetes (Nowotny et al., 2015; Lima Júnior et al., 2021), inflammation-related problems (Li et al., 2012), and miscarriage (Liang et al., 2020; Agarwal et al., 2012). Traditional medicinal therapies for such problems include the use of this plant.

Extracts of the leaves and pseudobulbs of *P. karwinskii* were able to inhibit ROS production. This could explain the antidiabetic properties of orchid leaves and pseudobulbs in traditional medicine. This is because increased glucose levels auto-oxidize glucose and increase the production of free radicals and ROS (Lotfi et al., 2020), which increase oxidative stress in β-cells, leading to insulin resistance and decreased secretion of this hormone (Karunakaran and Park, 2013; Sheweita et al., 2020; Lima Júnior et al., 2021). In addition, elevated blood glucose levels increase ROS production and the formation of advanced glycation end products, which are factors involved in the development of diabetes mellitus (Nowotny et al., 2015; Lima Júnior et al., 2021).

The use of the plant to treat inflammation-related conditions could also be due to their ability to inhibit ROS, as oxidative stress can cause inflammation by increasing the generation of proinflammatory mediators, such as TNF-α and iNOS (Li et al., 2012). Previously, the ability of *P. karwinskii* leaves extract to decrease inflammation and inhibit ROS formation has been corroborated (Barragán-Zárate et al., 2020).

The ability of *P. karwinskii* flowers extract to inhibit ROS could be related to the medicinal use of this part of the plant in the prevention of abortion. Oxidative stress is a contributing factor to miscarriage (Liang et al., 2020; Agarwal et al., 2012) and the antioxidant defense system of enzymes, such as glutathione peroxidase (GSH-Px), is decreased in women with this condition (Zachara et al., 2001; Omeljaniuk et al., 2015). Furthermore, the addition of gold to the infusion of flowers could improve their antioxidant properties, decrease oxidative stress, and help prevent miscarriage. This is because gold compounds can inhibit the production of ROS (Cuevas et al., 2012) and gold nanoparticles can enhance the levels of antioxidant enzymes, such as SOD, CAT, GSH-Px, and GSH (Muller et al., 2017).

The antioxidant compounds identified in *P. karwinskii* are rutin (Yeh et al., 2014), kaempferol-3-O-rutinoside (Wang et al., 2015), embelin (Gupta et al., 2012), and guanosine (Quincozes-Santos et al., 2014; Courtes et al., 2019). These compounds decrease oxidative stress by increasing the antioxidant activity of SOD, CAT, GSH-Px, and GSH. Azelaic acid, also found in *P. karwinskii*, reduces ROS generation in neutrophils (Akamatsu et al., 1991). Rutin and azelaic acid are present in both leaves and pseudobulbs, and the plant parts have the highest ROS inhibition ability. Embelin and kaempferol-3-O-rutinoside are present only in the leaves, the plant part with the highest inhibition of ROS and DPPH radicals. Guanosine is present in both the leaves and flowers of plants. In the chromatograms (Figures 1, 2, and 3), it was observed that the leaves extract presented a higher intensity of peaks corresponding to compounds with antioxidant capacity, followed by pseudobulbs and flowers extracts, which agrees with the results of antioxidant capacity, both in cells and *in vitro*.

## 5 Conclusion

The compounds present in each part of the *P. karwinskii* orchid were identified. Based on a review of the biological effect of the compounds evaluated in previous studies, it was found that there is a close relationship between the biological activities reported for these compounds and the traditional uses attributed to the different parts of the orchid in traditional medicine. The compounds unique to each part of the plant contribute to its medicinal properties, although these properties may also be enhanced by the common compounds they share.

According to the PCA analysis, the compounds with the highest contribution to the variance (kaempferol-3-O-ruthinoside, rutin, quinic acid, embelin, and chlorogenic acid) correspond to those present in the leaves, including some compounds exclusive to this part of the plant.

## Declarations

### Author contribution statement

Gabriela Soledad Barragán-Zarate: Conceived and designed the experiments; Performed the experiments; Analyzed and interpreted the data; Wrote the paper.

Luicita Lagunez-Rivera: Conceived and designed the experiments; Analyzed and interpreted the data; Wrote the paper.

Rodolfo Solano: Analyzed and interpreted the data; Wrote the paper.

Candy Carranza-Alvarez: Performed the experiments; Contributed reagents, materials, analysis tools or data.

Diego Manuel Hernández-Benavides: Performed the experiments; Analyzed and interpreted the data.

Gerard Vilarem: Contributed reagents, materials, analysis tools or data.

### Funding statement

The research was supported by CONACYT (Consejo Nacional de Ciencia y Tecnología) project 270428, and the Instituto Politécnico Nacional projects SIP-2016RE/50, SIP-2128, SIP-20202023, SIP-20210285 and SIP-20220649.

### Data availability statement

Data included in article/supp. material/referenced in article.

### Declaration of interest’s statement

The authors declare no conflict of interest.

### Additional information

No additional information is available for this paper.
